# Baseline and Strategic Effects behind Mindful Emotion Regulation: Behavioral and Physiological Investigation

**DOI:** 10.1371/journal.pone.0116541

**Published:** 2015-01-15

**Authors:** Alessandro Grecucci, Nicola De Pisapia, Derangala Kusalagnana Thero, Maria Paola Paladino, Paola Venuti, Remo Job

**Affiliations:** Department of Psychology and Cognitive Sciences, University of Trento, Rovereto, TN, Italy; Cesena, ITALY

## Abstract

One of the consequences of extensive mindfulness practice is a reduction of anxiety and depression, but also a capacity to regulate negative emotions. In this study, we explored four key questions concerning mindfulness training: (1) What are the processes by which mindfulness regulates our emotions? (2) Can mindfulness be applied to social emotions? (3) Does mindfulness training affect emotionally driven behavior towards others? (4) Does mindfulness alter physiological reactivity? To address these questions, we tested, in two experiments, the ability of mindfulness meditators to regulate interpersonal emotions (Experiment 1) and interactive behaviors (Experiment 2) as compared to naïve controls. To better understand the mechanisms by which mindfulness regulates emotions, we asked participants to apply two strategies: a cognitive strategy (mentalizing, a form of reappraisal focused on the intentions of others) and an experiential strategy derived from mindfulness principles (mindful detachment). Both groups were able to regulate interpersonal emotions by means of cognitive (mentalizing) and experiential (mindful detachment) strategies. In Experiment 1, a simple effect of meditation, independent from the implementation of the strategies, resulted in reduced emotional and physiological reactivity, as well as in increased pleasantness for meditators when compared to controls, providing evidence of baseline regulation. In Experiment 2, one visible effect of the strategy was that meditators outperformed controls in the experiential (mindful detachment) but not in the cognitive (mentalize) strategy, showing stronger modulation of their interactive behavior (less punishments) and providing evidence of a strategic behavioral regulation. Based on these results, we suggest that mindfulness can influence interpersonal emotional reactions through an experiential mechanism, both at a baseline level and a strategic level, thereby altering the subjective and physiological perception of emotions, but also biasing interactive social behavior.

## Introduction

In the last twenty years, several studies have shown that mindfulness meditation is associated with increased well-being and emotional balance [1]. Mindfulness is usually defined as “a kind of nonelaborative, nonjudgmental, present-centered awareness in which each thought, feeling, or sensation that arises in the attentional field is acknowledged and accepted as it is” [2]. The potential of mindfulness has been recognized also in the several types of clinical interventions. Indeed, mindfulness has been incorporated in models of psychotherapy such as the well-known Mindfulness-Based Stress Reduction [3], Mindfulness Based Cognitive Therapy [4], Dialectical Behavior Therapy [5], Acceptance and Commitment therapy [6], Psychoanalysis [7], and Intensive Short-Term Dynamic Psychotherapy [8], proving its ability to increase distress tolerance and regulate symptoms such as anxiety [9, 10], depression [11, 12] and psychological stress [13].

Although the advantages of mindfulness seem well established, the mechanisms by which this type of meditation influences emotions are still under investigation. Recent theoretical accounts were put forward in order to decompose what lies behind mindfulness meditation. As some authors have recently proposed, mindfulness may enhance emotion regulation abilities. About this point, Bishop [2] speculates that mindfulness may promote an objective and adaptive way of responding to emotional triggers, in contrast to dysfunctional and automatic patterns of emotional reactions. However, only a few studies have examined the effects of mindfulness on emotional reactivity [14]. The results from these studies are very promising, suggesting that mindful training can improve emotion regulation. For example, mindfulness training induced a reduction of emotional interference [15], decreased negative mood states [16], improved positive moods [17], decreased skin conductance reactivity [8], and reduced amygdala activity [19].

One recent experiment tested the ability of mindfulness meditators to reduce the perceived intensity of simple unpleasant pictures when attending to the stimuli in a state of mindful awareness [20]. The authors tested twelve experienced and ten beginner meditators who viewed pleasant, unpleasant, and neutral pictures while undergoing a functional Magnetic Resonance Imaging (fMRI) scanning. Results showed that both groups were able to experience reduced emotional reactions at subjective and neural level when asked to attend to the pictures in a mindfulness awareness state. However, these results were not compared to a control group (non-meditators). A similar result, but with emotional sounds, was obtained by Brefczynski-Lewis and colleagues [21], who found in expert and novice meditators a negative correlation between hours of meditation experience and right amygdala activation. Other experiments showed that mindfulness practiced after an experimentally induced sad mood reduces dysphoric mood states [22].

However, even though partial evidence of a mindful emotion regulation was provided, a fundamental question remains unsolved: what are the mechanisms leading mindfulness practitioners to positively influence their emotional reactions? Holzel et al. [23] suggested five potential mechanisms through which mindfulness practice might improve psychological well-being. These mechanisms are: 1) attention regulation, consisting in an increased attentional ability to maintain the focus on a specific object rather than being distracted by negative emotional stimuli (thoughts, images, behavioral tendencies, etc..); 2) body awareness, consisting in becoming aware of bodily sensations and in the ability to remain focused on them; 3) emotion regulation through changing the negative interpretation of events (reappraisal), as well as through exposure-extinction-reconsolidation, and through letting emotions affect the individual without reacting to them; and 4) change in perspective on the self, promoting a detached but aware perspective over oneself and external reality. Of particular importance for the goal of the present research, Holzel [23] proposed that mindfulness practice might improve emotion regulation. Also, Garland et al. [24] hypothesized that mindfulness may act upon emotions by increasing positive reappraisals of the situations, a process through which negative events are reframed in a more benign and positive meaning. Moreover, Modinos et al. [25] showed that mindfulness dispositional traits were positively correlated with the activation of the dorsomedial prefrontal cortex during reappraisal, thus establishing a link between mindfulness and reappraisal.

However, other studies showed opposite results, namely reduced reappraisal activity in mindfulness meditators. For example, some authors reported reduced cognitive control and prefrontal activity, as well as increased bottom-up sensory processing [26, 27]. As Holzel et al. [23] argued, these puzzling data pose the question of whether mindfulness produces emotion regulation through cognitive reappraisal or at a more experiential level (e.g., mechanism 4 in [23] account).

According to some authors [2, 3] and classical definitions in the field, mindfulness practice increases the ability to create a detached perspective or a psychological distance from mental events by adopting a detached and acceptance based perspective. For this reason, mindfulness may be considered an experiential strategy rather than a cognitive strategy. It does not change the way we interpret a situation, rather it changes how we experience the situation. In other words, mindfulness meditators learn to adopt a decentered view of inner and external reality [28].

Based on these considerations, one may ask whether mindfulness is able to influence our emotions because it changes the way we cognitively reappraise the situations (“how we perceive the events”), or because it gives a detached and acceptance based perspective over them (“how we experience the events”).

To address this issue (Goal 1), in the present research we tested the contribution of these mechanisms in two experiments. Mindfulness meditators and controls were confronted with partner’s selfish or altruistic behaviors and asked to report their emotional reactions (subjective ratings and physiological responses in Experiment 1) and their emotionally driven behaviors (rejection rates in Experiment 2). In addition they were asked to implement, one at a time, two emotion regulation strategies (“mentalizing” or interpersonal reappraisal, and “mindful detachment”), so we could test the possibility that the positive effects of mindfulness on emotional and behavioral reactions are related to the improved ability to cognitively reappraise and/or mentally detach oneself from events. Finally, to control for the possibility that mindfulness meditators might have acquired a non-specific improvement in emotion regulation, we also added a “look and respond” condition, in which no emotional regulation strategies were used.

If emotion regulation in mindfulness relies upon a cognitive mechanism, meditators would show reduced emotional reactions when applying the reappraisal (mentalizing) strategy, whereas, if the experiential hypothesis is true, they may show larger differences when applying the mindful detachment strategy. Finally, an alternative hypothesis can be advanced of no difference arising between meditators and controls in the cognitive or experiential strategy, but only a difference in the emotional baseline.

In the present study, we also asked whether mindfulness meditators are able to influence social emotions and socially-driven behaviors. A previous experiment showed partial evidence that mindfulness was able to reduce the emotional perception of non-social emotional stimuli [20]. To this aim (Goal 2 and 3), we used social emotions stemming from interpersonal situations to test the effect of mindfulness training on both the perception (Experiment 1) and the interactive behavior (Experiment 2). Following recent advances in the field of social emotion regulation [29–31], we used, for the first time, interpersonal interactive situations that offer the possibility of studying social emotions (such as anger aroused by the unfair treatment of a partner with whom we are interacting), rather than using non-social emotions, as previous studies in the field have [20]. We predict that mindfulness meditators, when compared to controls, exhibit a higher capacity to regulate their social emotions and their social behaviors.

Last but not least, we recorded SC while performing Experiment 1, to see whether meditators relative to controls have different physiological reactivity due to their mindfulness training (Goal 4). Previous studies showed mixed results about the effect of mindfulness over physiological reactivity when perceiving non-social emotions (see [32] for a positive result and [33] for a null result).

Notably, in the present study, we did not want to test for changes in the pre and post training phase. The main aim was to explore differences in the use of strategies between meditators and controls as an effect of prolonged mindfulness training. Importantly, we also used a baseline condition to verify whether differences between the two groups were present independently of the strategies adopted, and thus, also independently of the mindfulness training.

## Experiment 1: Mindfulness and Social Emotion Regulation

### Methods

The ethical review board of the University of Trento approved the study and written informed consent was obtained from all participants.

**Participants.** In the first experiment, we tested 18 experienced meditators and 26 control participants (with no experience in mindfulness training). The mean age of the participants was 35.11 (SD ± 12.75) years and their mean education was 18.16 (SD ± 3.68) years long. Control participants were recruited from the local population and were naïve to any meditation practice. The mean age of the participants was 36.92 (SD ± 13.87) years, and their mean education was of 17.34 (SD ± 5.16) years long. The study was approved by the University of Trento Ethical Committee and all participants were asked to read and sign an informed consent, as provided in the code of ethics for research. Both groups (meditators and controls) had no previous neurological or psychiatric problems. There were no significant differences between the groups with respect to age (p>0.05), education (p>0.05), and gender (p>0.05).

**Mindfulness Training.** Participants had received a formal training in mindfulness meditation by an expert Buddhist teacher (Rev. D. Kusalagnana, one of the authors) at a Meditation Center in Bosentino, Italy. The minimum period of training was four weeks. Every session lasted for about one hour and a half, in which participants performed sitting meditation sessions. Every session started with a mindfulness of breathing training and ended with bodily awareness training.

The basic procedure was as follows: the practitioner maintains an upright sitting posture, either in a chair or cross-legged on the floor and he/she attempts to maintain attention on a particular focus, most commonly on the somatic sensations of his or her own breathing. Whenever attention wanders from the breath to inevitable thoughts and feelings that arise, the practitioner will simply take notice of them and then let them go, as attention is gently returned to the breath. This process is repeated each time attention wanders from the breath. As sitting mindfulness is practiced, there is an emphasis on simply taking notice of whatever the mind happens to wander to and accepting each object (i.e. thoughts, feelings, sensations, environmental stimuli etc.) without making judgments about it or elaborating on its implications, additional meanings, or need for action [2, 4, 34]. The practitioner is further encouraged to use the same general approach outside of his or her formal mindfulness practice as much as possible by bringing awareness back to the here-and-now during the course of the day, with or without using breath as an anchor, whenever he or she notices a general lack of awareness or his or her attention becoming focused on streams of thoughts, worries, or ruminations [2]. The training was delivered on a one day per week basis. Between sessions, participants were asked to practice on a daily basis.

### Experiment 1: Experimental Procedure

**Figure 1:**
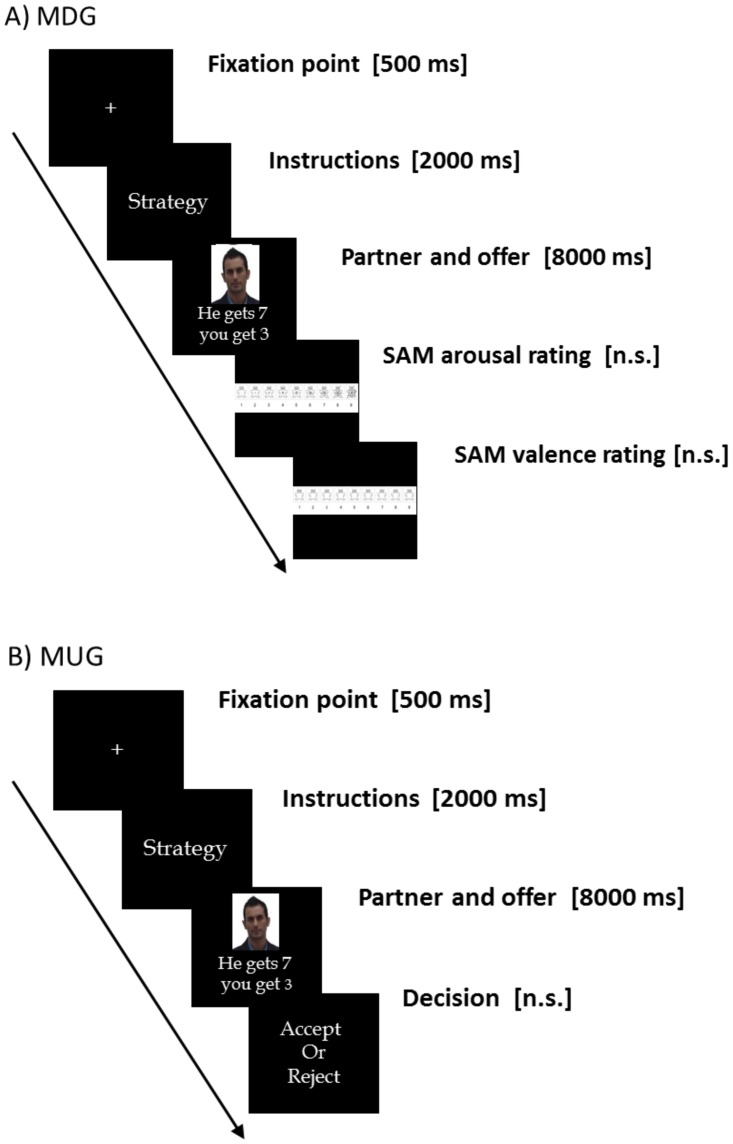
Timelines for the two Experiments. A) Experiment 1, the sequence of events in the Modified Dictator Game. B) Experiment 2, the sequence of the events in the Modified Ultimatum Game.

Both groups took part in a Modified version of the Dictator Game (MDG) [30, 31]. After providing informed consent, participants were first instructed as to the rules of the MDG. Even though the MDG is derived from economic psychology, it was used in the present context only to elicit interpersonal (negative and neutral) emotions (i.e. we were not interested in exploring behavioral economics effects as such). In the game, there are two players: the first player is defined as “dictator” and the second player is the “receiver.” In this modified version, participants played as receivers while applying emotion regulation strategies (described below). The dictator decided how to split ten euros between him/herself and the receiver. Five different types of (receiver:dictator) offers were possible: 1€:9€, 2€:8€, 3€:7€, 4€:6€ and 5€:5€, repeated 4 times for a total of 20 offers for each of the three experimental conditions (looking, mentalizing, and mindful detachment). The receiver was asked to passively observe and then evaluate the emotions elicited by the altruistic or selfish behavior on a two-part emotional scale (one for arousal and one for valence using a visual analog scale known as the Self-Assessment Manikin, [35]). The association of offers and the faces of partners were completely randomized. During the instructions, it was emphasized that the offers were real and previously recorded from an actual partner (the ones whose face was presented at the beginning of each round) (see Fig. 1A for a timeline). Importantly, the MDG includes in its use an additional manipulation of the emotion regulation strategy. A written protocol was given to participants describing both strategies. The cognitive strategy of mentalizing required participants to “reinterpret the intentions of the player as less negative.” The protocol included the presentation of an image depicting a crying woman. Participants were told that this event can be interpreted in various ways; for example, one may think that the woman is suffering from bereavement or has a slight headache. Both interpretations are appropriate, but their effect is different: the first interpretation increases the negativity of the event, the second one decreases it. Participants were then asked to always reinterpret the events as less negative. They were then given the instructions of the MDG and told to focus on the mind of the player in order to build an interpretation of his/her intentions as less negative. The second strategy, named mindful detachment, is a less cognitive and more experiential strategy. In line with one of the mindfulness principles, participants were asked to observe the events happening during the game and adopt a detached perspective with an attitude of acceptance and lack of judgment. Again, these instructions were written and a picture was then presented depicting a scene of war and terrorism. Participants were told that the ways in which we experience the situation determines how we feel about it. Then participants were told how to apply this strategy to the context of the MDG (assume a detached, non-judgmental, and acceptance based perspective on players’ behavior). Finally, in the control condition (looking), participants were asked to respond as spontaneously as possible. Before the experiment, participants performed two training trials, in which the experimenter made sure everybody understood the rules of the game and were able to apply the emotion regulation strategies.

After the experiment, participants were asked to fill out some self-administered questionnaires: PANAS (Positive Affect and Negative Affect Scales, [36]) to assess the positive and negative affective states; ERQ (Emotional Regulation Questionnaire, [37]), to assess two basic emotion regulation strategies, reappraisal and suppression; and CERQ (Cognitive Emotion Regulation Questionnaire, [38]), to assess several cognitive regulatory strategies.

Additionally, meditators were asked to provide all the relevant information about their meditation practice (starting date, frequency, daily practice, length of sessions).

**Galvanic response recording.** A BIOPAC MP100 system was used to record skin conductance (SC). The AcqKnowledge software provided with the BIOPAC system was used to record skin conductance (Biopac Systems, Goleta, CA). The sampling frequency of skin conductance responses was sampled at 1000 Hz with a constant voltage of 0.5 V. For analysis of galvanic response data, Ledalab, a Matlab-based toolbox, was used. Data were down-sampled at 100 Hz, the artifact was manually corrected, and then it was optimized by the Ledalab algorithm. The Continuous Decomposition Analysis (CDA) was performed comprising a decomposition of SC data into continuous signals of phasic and tonic activity [39]. Standard statistical analyses were applied to global mean data. Notably, we were not interested in single trial responses or in following previous studies [40], but considered SC as a global measure of the implementation of the strategy (looking, mentalizing, and mindful detachment).

### Experiment 1: Results

**A) Behavioral Results.** To begin with, in order to assess if the two groups were able to understand and apply the strategies (i.e. the strategies had an effect on them), we computed separate ANOVAS for each group for both Arousal and Valence ratings as the dependent variable, with the strategies (looking, mentalizing, mindful detachment) and offer amounts (€5, €4, €3, €2, €1) as the within-subject factors.

For the control group, the strategy (F(2,50) = 28.530, p<0.0001) and the offer (F(4,100) = 4.055, p<0.005) had a significant effect, but the interaction (F(8,200) = 0.912 p = 0.507) for arousal did not; the effect of the strategy (F(2,50) = 25.308, p<0.0001) and the offer (F(4,100) = 48.865, p<0.0001), as well as the interaction (F(8,200) = 12.169 p<0.0001) for valence, were also significant. Exploring the interaction in the valence ratings, corrected post hoc for multiple comparisons, revealed significant differences for all offers (p<0.003) when comparing the mentalizing strategy with the look condition and for offer 1 and 5 when considering the comparison between detaching and looking (p<0.003). Finally, when comparing the two strategies, all the offers resulted in significant differences (p<0.003). These results replicate previous findings [30].

For the meditation group, a significant effect of strategy (F(2,34) = 25.577, p<0.01) and of offers (F(4,68) = 2.785, p<0.05) emerged, but not related to the interaction (F(8,136) = 0.874 p = 0.540) for arousal. A significant effect of strategy (F(2,34) = 3.387, p<0.05) and of offers (F(4,68) = 24.078, p<0.0001) also arose, as well as the interaction (F(8,200) = 3.670 p<0.001) for valence. Exploring the interaction in the valence ratings, corrected post hoc for multiple comparisons, revealed significant differences for offer 1 and 3 (p<0.003) when comparing the mentalizing strategy with the looking condition. No other effects reached statistical significance.

To examine the differences between the meditators and the controls, two mixed ANOVAs were computed: one for valence and one for arousal ratings as dependent variables, with the strategies (looking, mentalizing, mindful detachment) and offer amounts (€5, €4, €3, €2, €1) as within-subject factors, and the group type (meditators vs. controls) as a between-subject factor.

**Figure 2:**
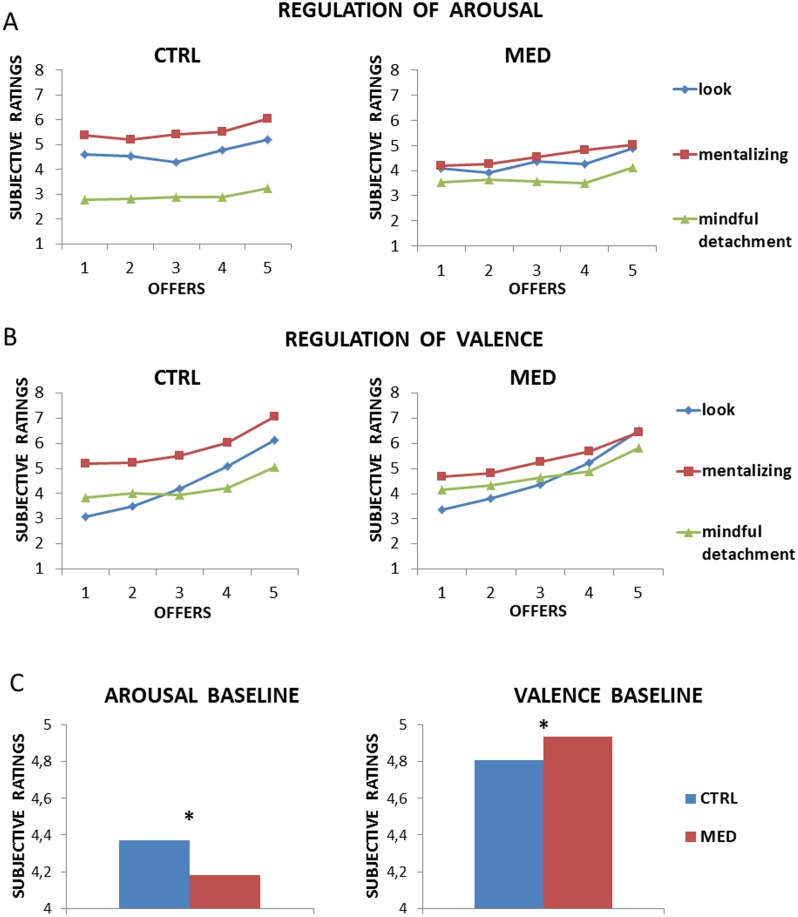
Results from Experiment 1. Both groups were able to regulate their emotions in terms of arousal (Panel A) and valence (Panel B). Collapsing for offers and strategies, meditators showed a lesser emotional reactivity (lower arousal, Panel C, left) and more pleasant emotions (higher valence, Panel C, right).

For the arousal ratings, a significant main effect of strategy (F(2,84) = 27.587, p<0.0001) and offer amount (F(4,168) = 6.541, p<0.0001) were obtained, as well as a significant strategy by group interaction (F(2,84) = 6.428, p<0.005). However, the offer by group interaction and the offer by strategy interaction were not significant (respectively, F(4,168) = 0.208, p = 0.934), F(8,168) = 0.936, p = 0.487) and neither was the triple interaction (F(8,336) = 0.876, p = 0.537). See Fig. 2A and Table 1. To explore the strategy by group interaction, we collapsed the offers and computed post hoc analyses corrected for multiple comparisons. However, statistics failed to reach a significant threshold corrected for multiple comparisons (all p>0.003). To test for the hypothesis that the two groups may have overall differences in experienced emotional strength, independently of the strategy used, we performed an independent t-test with the mean of ratings across all the conditions (offers and strategies) and found that meditators reacted less strongly (a lower arousal) when compared to controls (t(42) = -0.512, p<0.05). See Fig. 2C, left panel.

**Table 1:** Subjective ratings of Experiment 1.

**AROUSAL RATINGS**					
**LOOK**					
	*1€*	*2€*	*3€*	*4€*	*5€*
**CTRL**	4,58	4,54	4,29	4,76	5,20
**MED**	4,08	3,93	4,35	4,25	4,89
**MENTALIZE**					
	*1€*	*2€*	*3€*	*4€*	*5€*
**CTRL**	5,36	5,20	5,39	5,52	6,05
**MED**	4,20	4,26	4,55	4,80	5,02
**MINDFUL DETACHMENT**					
	*1€*	*2€*	*3€*	*4€*	*5€*
**CTRL**	2,76	2,81	2,89	2,89	3,23
**MED**	3,54	3,64	3,56	3,48	4,12
**VALENCE RATINGS**					
**LOOK**					
	*1€*	*2€*	*3€*	*4€*	*5€*
**CTRL**	3,06	3,48	4,19	5,09	6,13
**MED**	3,37	3,81	4,37	5,22	6,51
**MENTALIZE**					
	*1€*	*2€*	*3€*	*4€*	*5€*
**CTRL**	5,21	5,23	5,51	6,01	7,05
**MED**	4,66	4,80	5,26	5,66	6,45
**MINDFUL DETACHMENT**					
	*1€*	*2€*	*3€*	*4€*	*5€*
**CTRL**	3,85	4,02	3,93	4,21	5,03
**MED**	4,15	4,33	4,65	4,87	5,81

For valence, there was a significant main effect of strategy (F(2,84) = 21.588, p<0.0001) and offer amount (F(4,168) = 68.394, p<0.0001), as well as a significant strategy by group interaction (F(2,84) = 3.615, p<.05) and offer by strategy interaction (F(8,168) = 13.323, p<0.0001). However, the offer by group interaction was not significant (F(4,168) = 0.193, p = 0.942) and neither was the triple interaction (F(8,336) = 1.094, p = 0.367). See Fig. 2B and Table 1. To explore the strategy by group interaction, we collapsed for offers and computed independent sample t-tests. However, statistics failed to reach significance for every contrast (all p>0.003). To test for the hypothesis that the two groups may have overall differences in perceived valence, independently of the strategy used, we computed an independent t-test for the mean of ratings across all the conditions and found that meditators perceived the offers as more pleasant (higher valence) when compared to controls (t(42) = 0.128, p<0.05). See Fig. 2C, right panel.

**B) Questionnaire Results.** Subsequently, we tested for eventual differences in the use of cognitive regulation strategies (ERQ and CERQ). The two groups did not differ in terms of cognitive strategies (all p>0.05 for both ERQ and CERQ subscales). In a similar fashion, the two groups did not differ when considering stable emotional traits (PANAS, both positive affectivity and negative affectivity scales, p>0.05).

**Figure 3:**
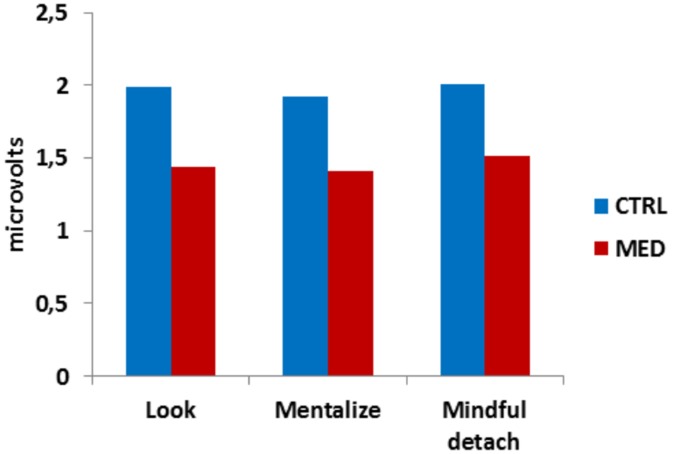
Physiological results. Skin conductance analyses confirmed a baseline difference between meditators and controls in terms of reduced physiological reactivity.

**C) Physiological Results.** Two subjects were excluded from the analyses because of technical problems during the recording of the data. To analyze the galvanic response, we selected a time window of interest from 1 to 5 seconds from the onset of the stimulus to be regulated (interpersonal situation). Independent sample t-tests showed that the two groups differed in the looking condition (t(49) = 2.217, p<0.05) and showed a trend during the implementation of the other two strategies (mentalizing, t(40) = 1.983, p = 0.054; mindful detachment t(40) = 1.791 p = 0.081)), see Fig. 3).

## Experiment 2: Mindfulness and the Regulation of Social Behavior

The ethical review board of the University of Trento approved the study and written informed consent was obtained from all participants.

### Experiment 2: Procedure

Participants played a Modified version of the Ultimatum Game (MUG). Six control subjects of Experiment 1 did not completed Experiment 2 and were substituted by new subjects. Again, the new sample did not statistically differ when compared to meditators (all p>0.05).

After providing informed consent, participants were first instructed as to the rules of the MUG. In line with the first experiment, present study used an economic game, not to study decision making as such, but to have an interactive game in which participants could experience interpersonal driven emotions and react to them. The game involves the division of 10€. The first player (Proposer) is free to formulate a proposal for a division of this sum of money while the second player (Responder) can only accept or reject the received proposal. If he/she rejects the offer, both players receive nothing. If he/she accepts, the sum of money is allocated between the two players as decided by the proponent. A plethora of studies have shown that when receiving an offer, an emotion is elicited in the player [41]. The nature of the emotions (valence and strength) depends on the quality of the offer. Unfair divisions typically elicit unpleasant emotions (such as anger, moral disgust, and disappointment), while fair divisions elicit pleasant emotions (happiness but also surprise). See [29–31] for a discussion on this point.

Similarly to Experiment 1, there were five possible monetary (proposer: responder) divisions (1€:9€, 2€:8€, 3€:7€, 4€:6€, and 5€:5€). Importantly, in the MUG, participants could accept or reject the offers. Previous studies show that rejection behavior is a way to punish the proposer for his or her unfairness [42]. This point is crucial to the present study, because it allows the game to be used for testing the effect of emotion regulation at a behavioral level and not only at a subjective level (as in Experiment 1 and the majority of emotion regulation studies, see [43] for a review). The percentage of rejections can be considered as an indirect and much more reliable index of having regulated the emotions elicited during the economic transaction. The same three regulatory conditions were given in the present experiment: looking, mentalizing, and mindful detachment. Twenty rounds for each condition were presented, each with 4 repetitions of the five possible divisions (see Fig. 1B for a timeline). The same protocol and procedure adopted in Experiment 1 was used.

### Experiment 2: Results

**A) Behavioral Results.** At first, we ran separate analyses on the two groups to be sure that participants understood the rules of the game and how to apply the strategies, with the percentage of offers rejected as the dependent variable, and strategies (looking, mentalizing, mindful detachment) and offer amount (€5, €4, €3, €2, €1) as within-subject factors.

**Figure 4:**
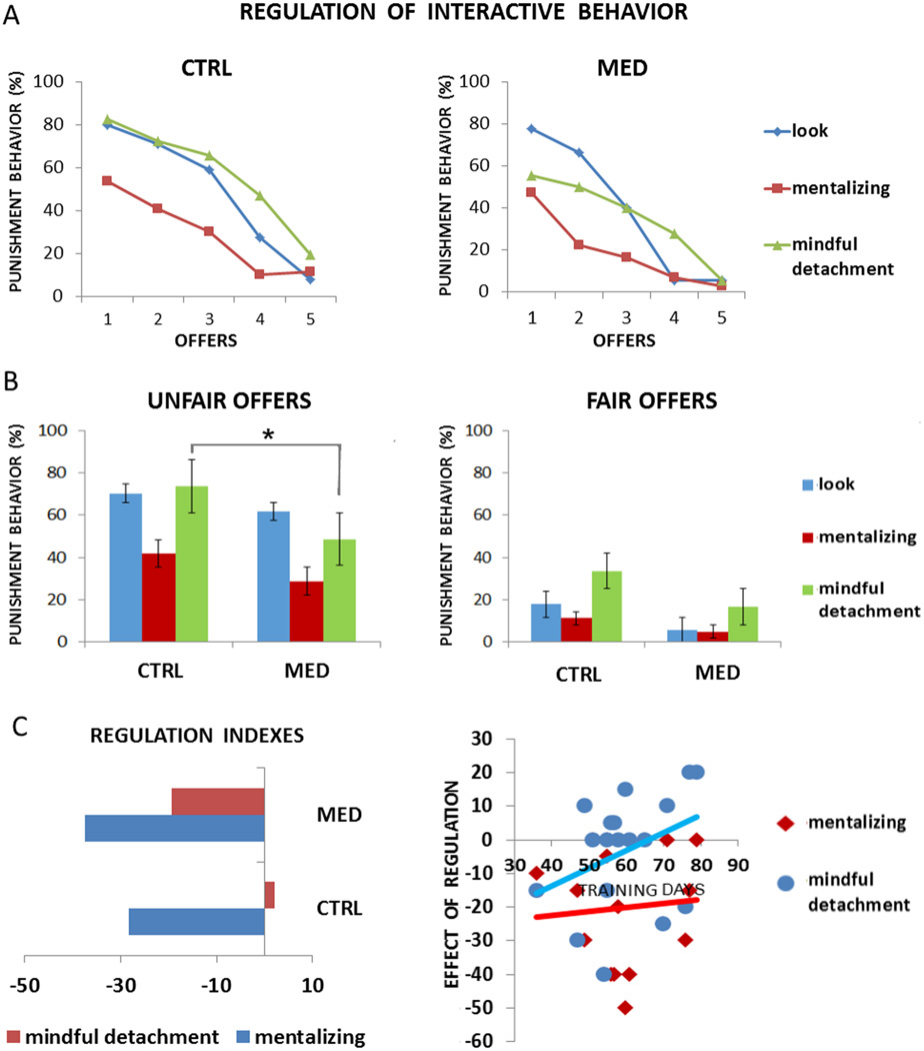
Results from Experiment 2. Both groups were able to apply the regulatory strategies. However, meditators outperformed controls when applying the experiential strategy (mindful detachment) (Panel A, B). Notably, the duration of training correlated with the ability to apply the experiential strategy (Panel C), thus confirming the effect of experiential rather than cognitive strategy as an effect of mindfulness practice.

Controls showed the significant effect of strategy and offer (respectively, F(2,50) = 19.886, p<.0001, F(4,100) = 72.636, p<.0001), as well as the interaction (F(8,200) = 5.654, p<.0001). To explore the interaction, post hoc analysis, corrected for multiple comparisons, was computed. This showed significant differences between looking and mentalizing for offers 1€, 2€, 3€ (p<0.003), between looking and mindful detachment for offer 4€ (p<0.003), and between mentalizing and mindful detachment for offers 1€, 2€, 3€, 4€ (p<0.003). See Fig. 4A and Table 2.

**Table 2:** Rejection rates of Experiment 2.

**LOOK**						
	*1€*	*2€*	*3€*	*4€*	*5€*	
**CTRL**	80,26	71,05	59,21	27,63	7,89	
**MED**	77,77	66,66	40,27	5,55	5,55	
**MENTALIZE**						
	*1€*	*2€*	*3€*	*4€*	*5€*	
**CTRL**	53,94	40,78	30,26	10,52	11,84	
**MED**	47,22	22,22	16,66	6,94	2,77	
**MINDFUL DETACHMENT**						
	*1€*	*2€*	*3€*	*4€*	*5€*	
**CTRL**	82,89	72,36	65,78	47,36	19,73	
**MED**	55,55	50	40,27	27,77	5,55	

The meditator group showed a significant effect of strategy and offers (respectively, F(2,34) = 10.979, p<.0001, F(4,68) = 28.270, p<.0001), as well as the interaction (F(8,200) = 4.879, p<.0001). To explore the interaction, post hoc analysis, corrected for multiple comparisons, were computed. This showed significant differences between looking and mentalizing for offer 2€ (p<0.003), trends for offer 1€ and 3€ (p = 0.006, p = 0.004), and a significant difference between mentalizing and mindful detachment for offer 3€ (p = 0.004). These results are in line with previous findings (see Grecucci et al. 2013a). See Fig. 4A and Table 2.

To examine the differences between meditators and controls in their interactive behavior, a mixed ANOVA was computed with percentage of offers rejected as the dependent variable, strategies (looking, mentalizing, and mindful detachment) and offer amounts (€5, €4, €3, €2, €1) as within-subject factors, and group type (meditators vs. controls) as a between-subject factor.

Significant main effects of strategy (F(2,84) = 24.954, p<.0001), offer amount (F(4,168) = 90.421, p<.0001), as well as a significant strategy by group interaction (F(2,84) = 3.500, p<.005) and by offer (F(2,336) = 9.530, p<.0001), were obtained. However, offer by group interaction was not significant (F(4,168) = 1.075, p = 0.370) and neither was the triple interaction (F(8,336) = 0.723, p = 0.671). To explore the strategy by group interaction, we collapsed for all offers and computed post hoc analyses, corrected for multiple comparisons. Only the interactive behavior when applying the mindful detachment strategy resulted in a significant effect (p<0.01) (not shown in Fig. 4). Separating for unfair and fair offers, we found that the effect was still significant for unfair offers (1 & 2, p<0.008) but not for fair offers (4 & 5, p>0.008). Thus, the test confirmed a selective effect of mindfulness meditation on unpleasant emotions. See Fig. 4B. To make more explicit the regulatory abilities of both groups, two indexes were additionally calculated: the difference between both strategies (mentalizing and mindful detachment) and the control condition looking, collapsed for all offers. See Fig. 4C, left panel. Notably, days of practice correlated with the mindful detachment index (rho = .425, p<.05), but not with the mentalizing index (rho = .065 p = .399). This further confirms the link between meditation practice and the ability to apply the experiential strategy (mindful detachment) rather than the cognitive strategy (mentalizing). See Fig. 4C, right panel.

**B) Questionnaire Results**. Then we tested for eventual differences in the usage of cognitive regulation strategies (ERQ and CERQ). The two groups did not differ in terms of cognitive strategies (all p>0.05 for both ERQ and CERQ subscales), further confirming that mindfulness meditation does not act upon cognitive regulation strategies. In a similar fashion, the two groups did not differ when considering stable emotional traits (PANAS, both positive affectivity and negative affectivity scales, p>0.05).

## Discussion

In this study we performed two experiments to compare the emotional reactivity and interaction patterns of a group of meditators while adopting cognitive and experiential strategies against a group of non-meditators.

In Experiment 1 (MDG), both meditators and controls were able to apply the cognitive and experiential strategies and had their emotions properly regulated, as shown by individual group analyses. Arousal for both groups had a monotonous behavior (independently of the level of selfishness of the proposer), whereas valence showed, additionally, an interaction between types of behavior and strategies applied. These results replicate previous findings (see [30] for a detailed discussion). More interestingly, individuals trained for mindfulness meditation differed from control participants for the overall strength in emotional reactivity (lower arousal) and the perceived pleasantness of the situation (higher valence). This difference may be due to the long term effects of the received training and not the implementation of the specific strategies (no significant effect of strategies). Importantly, the two groups did not differ in the baseline looking condition, thus excluding possible group differences based on external factors other than the meditation training. Also, the two groups did not differ in terms of emotional traits (PANAS trait scores), confirming that the differences observed are not due to random group differences (that are independent of mindfulness training or of strategic regulatory skills), but to the long lasting effect of mindfulness training. Notably, meditators were apparently less able to apply the strategies (less differences between the ratings when applying the strategies as compared to the looking condition). However, another interpretation, confirmed by analyses of the groups, is that meditators experienced less emotional reactions. This interpretation is also supported by the physiological data that reported less autonomic arousal in meditators when compared to controls. Importantly, this modulation is not due to the use of a specific regulatory strategy. In sum, this experiment was able to provide evidence that mindfulness meditation reduces social emotional reactions at the baseline level.

In Experiment 2 (MUG), participants were asked to apply emotion regulation strategies in a context that allowed us to detect the effect of regulatory mechanisms over explicit interactive behavior (rejection of unfair behaviors). Indeed, participants’ punishment behavior was used as an index to assess the effect of two different regulatory strategies over social interactions. Both groups showed independent effects of regulation of the two strategies over the control condition looking. Notably, meditators outperformed controls when applying the experiential strategy “mindful detachment,” which indeed is similar to what mindfulness training teaches. This confirms that as an effect of mindfulness training, participants develop an attitude of detachment from emotional experience and non-judgmental acceptance toward others’ behavior. In fact, when meditators were asked to apply this strategy, their punishment behavior was reduced. And this was especially true for unfair offers that usually elicit stronger reactions of anger and disgust in the receiver (see [29, 30] for this point).

A main goal of this study was to explore the mechanisms that allow mindfulness to work. In Experiment 1, we found evidence that mindfulness regulates emotions at a baseline level (independently of the strategy and of the offers used). However, in Experiment 2, we found evidence that, beside general effects of regulation, mindfulness increases a strategic regulation of socially driven behaviors. In other words, meditators differed from controls only when applying one of the two strategies, the experiential strategy (mindful detachment). They did not differ when using the cognitive strategy (mentalizing) nor in the baseline (look and respond) condition. This indicates that mindfulness acts upon the perspective one has in relation to the events and not through a cognitive modification of the meaning associated to the events.

Even though the efficacy of mindfulness on well-being seems established, the mechanisms behind meditation that influence emotions are still poorly understood. The authors put forward the notion that mindfulness may influence emotion regulation (for example, [2]). Experimental proofs show that mindfulness causes a reduction of emotional interference [15] and the intensity of unpleasant pictures [20]. However, the mechanisms by which mindfulness regulates emotion is unclear. Some authors have proposed that this may happen by cognitive regulation (reinterpreting the meaning of the situation through reappraisal abilities, see for example, [23, 24]) or by a more experiential regulation (developing a psychological distance, see for example, [2, 3]). To our knowledge, this is the first study that shows that mindfulness regulates emotions not through a cognitive regulation but by developing a stronger ability to create a distance from an emotional event (experiential mechanism). In other words, mindfulness helps to adopt a decentered and more distant view of inner and external reality [28].

A second goal of this study was to investigate the hypothesis that individuals who practice mindfulness meditation are able to regulate social emotions rather than non-social emotions. In Experiment 1, we found a confirmation that meditators demonstrated a reduced emotional reactivity (lower subjective and physiological arousal) and less unpleasant emotions (higher valence) in a social situation (a modified version of the Dictator Game), thus suggesting that individuals who practice mindfulness experience less severe social emotional reactions when treated selfishly by others.

A third goal of the study was to test whether mindfulness training can affect socially driven behaviors and not only the perception of social emotions. In Experiment 2, we found evidence that a socially driven behavior, such as the rejection of unfair exchanges, was affected by mindfulness training (reduced rejection rates by meditators when applying one particular strategy). As humans, we daily interact with others and emotions are constantly generated in a social interpersonal context. Knowing that mindfulness practice does affect our interpersonal reactions is of great importance, also, at a clinical level. The ability to regulate emotions is essential for healthy psychological functioning. Deficits in the regulation of interpersonal emotions have been linked to psychiatric disorders that involve heightened emotional experiences, such as Borderline Personality Disorder [5]. Understanding how patients experience and fail to regulate such interpersonal emotions is of fundamental importance. The cultivation of mindfulness may provide an effective tool to improve emotion regulation when interacting with others, especially for clinical populations characterized by strong emotional dysregulation [44].

A fourth goal was to assess the effect of mindfulness training at a physiological level. In Experiment 1, we found evidence that mindfulness training reduced physiological arousal (weaker galvanic skin response) in meditators as compared to control. This effect was independent from the strategy. Previous studies showed that females practicing meditation for the first time displayed a reduction of galvanic response, after meditation training, with changes enhanced among subjects with positive expectations [45]. Another study [32] showed significantly reduced SC during the meditative state and the following resting state in a MBSR treatment. Other studies showed decreased skin conductance reactivity [18] and reduced amygdala activity [19]. However, Erisman and Roemer [33] failed to find such a modulation of physiological activity. Our data support a baseline reduction of SC due to the meditation training. In the looking baseline condition, the difference between the two groups reaches a significant level, whereas, when applying the strategies, results did not reach significance even though two trends were visible. This may be due to the fact that implementing a regulatory strategy may be effortful, as a recent study showed [40]. Notably, previous studies recorded SC during meditations and not during an experimental task independent of the training. This may indicate that beside a reduction in the physiological activity during mindfulness, the effects on the nervous system are long lasting and independent of the meditation itself. This is of great importance if we consider that mindfulness can be applied to anxiety and depressive disorders in which physiological activity is usually higher relative to non-psychiatric populations. Future studies will evaluate the effect of mindfulness on physiological reactivity after the treatment period in normal and abnormal populations. Last but not least, Civai and colleagues [46] found that participants’ SC increased only when the Ultimatum Game was played for themselves and not for a third party. This finding suggests that participants’ negative emotional reaction in the Ultimatum Game is not elicited by unfairness of the offer tout-court but rather by the fact of being treated unfairly. In this line of reasoning, we can speculate that the effect of mindful detachment on the interactive behavior (that we observed in the present research) is due to a decrease in the intensity of self-related emotions.

## Conclusions

Recent research has suggested that emotion regulation may be improved through interventions based on mindfulness meditation training, which has been shown, for three decades, to have general health benefits [3, 13]. In particular, the ability to intentionally pay attention to present emotional experience with a non-judgmental attitude (i.e. non-reactive monitoring), cultivated through the practice of mindfulness, has been seen to change emotional reactivity, promoting a healthier “mindful emotion regulation” [47]. This study, for the first time, presents data that confirm the hypothesis of emotion regulation through mindfulness. These data also expand the field in the direction of showing, for the first time, that this practice can regulate social emotions and socially driven behaviors, thus paving the way for several practical applications in the field of mental health and social prevention.
